# Methodological Considerations in the Study of *Helicobacter pylori* Prevalence Among Geriatric Adults

**DOI:** 10.5152/tjg.2026.26218

**Published:** 2026-03-13

**Authors:** Sami Fidan, Arif Mansur Coşar

**Affiliations:** Department of Gastroenterology, Karadeniz Technical University Faculty of Medicine, Trabzon, Türkiye

To the Editor,

We read with great interest the article by Özden and Bulur titled “Prevalence of *Helicobacter pylori* Infection in Geriatric Adults: A Single-Center Cohort Study from Türkiye,” published in the *Turkish Journal of Gastroenterology*.^1^ The authors examined a large cohort of older adults and provided valuable data on the epidemiology of *Helicobacter pylori *(*H. pylori*) infection in a population that is relatively underrepresented in clinical studies. However, we believe that the findings should be interpreted with caution because of certain methodological limitations and selection bias.

The prevalence of *H. pylori* infection varies considerably across geographic regions and study designs and is generally higher in developing countries. In Türkiye, the population-based TURHEP study reported an overall prevalence of approximately 82.5%, with individuals aged 65 years and older found to be at relatively lower risk compared with younger groups.^2^ Nevertheless, population-based data focusing specifically on the geriatric population remain limited. The study by Özden and Bulur^1^ showed that although the prevalence of *H. pylori *infection slightly decreases with age, it remains high among older adults (56.3%). Although this finding provides valuable insight, the single-center, retrospective design limits generalizability and warrants careful methodological consideration. First, the study population comprised only patients undergoing upper gastrointestinal endoscopy. Patients at high risk of *H. pylori* infection, such as those with gastrointestinal bleeding, were also excluded. Such an endoscopy-based cohort typically includes individuals with gastrointestinal symptoms or those requiring clinical evaluation and, therefore, may differ substantially from the older population. This selection process may introduce selection bias and influence the observed prevalence of *H. pylori* infection. Second, in this study, the definition of *H. pylori *positivity required careful evaluation. Infection was defined as a positive stool antigen test result, histological detection of bacilli, or the presence of marked active gastritis despite a negative stool antigen test result. Discordant cases were adjudicated by consensus between the endoscopist and pathologist. Although this approach aims to resolve conflicting findings, attributing active gastritis to *H. pylori* infection without direct microbiological or histological confirmation may lead to subjective interpretation and increase the risk of misclassification.

Similarly, in our cohort of patients with dyspepsia, we observed a slight but statistically significant decrease in *H. pylori* positivity with increasing age, and the overall prevalence was 40.4%.^3^ This discrepancy illustrates how differences in diagnostic approaches and patient selection can influence prevalence estimates. In the current study, positivity was defined strictly by culture, rapid urease test results, or histopathological confirmation, whereas Özden and Bulur also considered active gastritis as a criterion for positivity. Such criteria may overestimate prevalence through misclassification because active gastritis can also result from drug-induced injury, bile reflux, or autoimmune gastritis.^4^

Another methodological concern relates to the potential impact of proton pump inhibitor (PPI) therapy on diagnostic accuracy. Current international guidelines emphasize that PPI therapy reduces the sensitivity of *H. pylori* tests and should be discontinued at least 2 weeks before testing.^5^ Given that polypharmacy is common among the geriatric population and PPI use is frequent, failure to account for recent PPI exposure may compromise diagnostic performance and consequently affect prevalence estimates.

From a clinical perspective, management of *H. pylori* infection in older adults requires individualized approaches because of multimorbidity and polypharmacy. Eradication therapy may improve life expectancy and quality of life through preventing gastric cancer and ulcer-related complications; however, antibiotic resistance, drug interactions, and adherence issues pose geriatric-specific challenges that must be considered in the risk–benefit assessment.^2^^,^^6^ Therefore, although the findings of the present study are valuable, recommendations for systematic screening and eradication should be approached with caution, and future multicenter, prospective studies are needed to clarify the feasibility of such strategies.

In summary, these methodological considerations highlight the challenges of accurately estimating *H. pylori* prevalence in older adults. The study by Özden and Bulur provides important data, but interpretation of the reported prevalence should take into account potential selection bias, diagnostic criteria, and factors such as medication use that may affect test performance. Future multicenter, prospective studies using standardized diagnostic approaches and population-based sampling strategies will help more clearly define the epidemiology of *H. pylori* infection in geriatric populations.

## Data Availability

The data that support the findings of this study are available on request from the corresponding author.

## References

[1] Özden Y Bulur O. Prevalence of *Helicobacter pylori* infection in geriatric adults: a single-center cohort study from Türkiye. Turk J Gastroenterol. 2026;37(3):380 386. (doi: 10.5152/tjg.2025.25499) 41846491 PMC12994429

[2] Ozaydin N Turkyilmaz SA Cali S. Prevalence and risk factors of *Helicobacter pylori* in Turkey: a nationally-representative, cross-sectional, screening with the ¹³C-Urea breath test. BMC Public Health. 2013;13:1215. (doi: 10.1186/1471-2458-13-1215) PMC3880349 24359515

[3] Erkut M Uzun DY Kaklıkkaya N Sociodemographic characteristics and clinical risk factors of *Helicobacter pylori* infection and antibiotic resistance in the eastern Black Sea region of Turkey. Turk J Gastroenterol. 2020;31(3):221 233. (doi: 10.5152/tjg.2020.18631) 32343234 PMC7197933

[4] Soykan İ Er RE Baykara Y Unraveling the mysteries of autoimmune gastritis. Turk J Gastroenterol. 2024;36(3):135 144. (doi: 10.5152/tjg.2024.24563) 39632655 PMC11899966

[5] Chey WD Howden CW Moss SF ACG clinical guideline: treatment of *Helicobacter pylori* infection. Am J Gastroenterol. 2024;119(9):1730 1753. (doi: 10.14309/ajg.0000000000002968) 39626064

[6] Kanat Unler G Erinanc OH Karakoca A Best treatment options for severe *Helicobacter pylori* infections. Turk J Gastroenterol. 2025;36(12):807 812. (doi: 10.5152/tjg.2025.24543) 41340331 PMC12684267

